# Machine Learning Algorithms to Predict Recurrence within 10 Years after Breast Cancer Surgery: A Prospective Cohort Study

**DOI:** 10.3390/cancers12123817

**Published:** 2020-12-17

**Authors:** Shi-Jer Lou, Ming-Feng Hou, Hong-Tai Chang, Chong-Chi Chiu, Hao-Hsien Lee, Shu-Chuan Jennifer Yeh, Hon-Yi Shi

**Affiliations:** 1 Graduate Institute of Technological and Vocational Education, National Pingtung University of Science and Technology, Pingtung 91201, Taiwan; lou@mail.npust.edu.tw; 2 College of Medicine, Kaohsiung Medical University, Kaohsiung 80708, Taiwan; mifeho@kmu.edu.tw; 3 Department of Surgery, Kaohsiung Medical University Hospital, Kaohsiung 80756, Taiwan; 4 Department of Surgery, Kaohsiung Municipal United Hospital, Kaohsiung 80457, Taiwan; hongtchang@gmail.com; 5 Department of General Surgery, E-Da Cancer Hospital, I-Shou University, Kaohsiung 82445, Taiwan; chiuchongchi@yahoo.com.tw; 6 School of Medicine, College of Medicine, I-Shou University, Kaohsiung 82445, Taiwan; 7 Department of General Surgery, Chi Mei Medical Center, Liouying, Tainan 73657, Taiwan; Hao_Hsien@hotmail.com; 8 Department of Healthcare Administration and Medical Informatics, Kaohsiung Medical University, Kaohsiung 80708, Taiwan; syeh@faculty.nsysu.edu.tw; 9 Department of Business Management, National Sun Yat-sen University, Kaohsiung 80424, Taiwan; 10 Department of Medical Research, Kaohsiung Medical University Hospital, Kaohsiung 80708, Taiwan; 11 Department of Medical Research, China Medical University Hospital, China Medical University, Taichung 40402, Taiwan

**Keywords:** breast cancer surgery, 10-year survival, machine learning, artificial neural network

## Abstract

**Simple Summary:**

No studies have discussed machine learning algorithms to predict recurrence within 10 years after breast cancer surgery. Artificial neural networks (ANN) model is superior to the other forecasting models in terms of accuracy in predicting recurrence within 10 years after breast cancer surgery. Surgeon volume was the best predictor of recurrence within 10 years after breast cancer surgery, followed by hospital volume and tumor stage. For patients who are candidates for breast cancer surgery or who have already received breast cancer surgery, these important predictors can also be used for education in the expected course of recovery and health outcomes. Integration of the machine learning algorithms applied in this study in other clinical decision-making tools would provide additional data that can be used to improve accuracy in predicting recurrence.

**Abstract:**

No studies have discussed machine learning algorithms to predict recurrence within 10 years after breast cancer surgery. This study purposed to compare the accuracy of forecasting models to predict recurrence within 10 years after breast cancer surgery and to identify significant predictors of recurrence. Registry data for breast cancer surgery patients were allocated to a training dataset (*n* = 798) for model development, a testing dataset (*n* = 171) for internal validation, and a validating dataset (*n* = 171) for external validation. Global sensitivity analysis was then performed to evaluate the significance of the selected predictors. Demographic characteristics, clinical characteristics, quality of care, and preoperative quality of life were significantly associated with recurrence within 10 years after breast cancer surgery (*p* < 0.05). Artificial neural networks had the highest prediction performance indices. Additionally, the surgeon volume was the best predictor of recurrence within 10 years after breast cancer surgery, followed by hospital volume and tumor stage. Accurate recurrence within 10 years prediction by machine learning algorithms may improve precision in managing patients after breast cancer surgery and improve understanding of risk factors for recurrence within 10 years after breast cancer surgery.

## 1. Introduction

Globally, breast cancer is the most commonly diagnosed cancer and the second leading cause of cancer-related death in women [1]. Currently, the main clinical approach is surgical treatment assisted with multi-disciplinary methods, such as radiotherapy, chemotherapy, and targeted therapy [2]. However, accurate prediction of recurrence after breast cancer surgery is associated with improved allocation and use of health care resources and can also improve coordination of healthcare and the efficiency of healthcare resource allocation for these patients.

Machine learning algorithms use sample data to learn and identify patterns so that they can use new data to make predictions. A recent study developed several novel artificial neural network (ANN) models for diagnosis of human colorectal cancer (CRC) based on data from The Cancer Genome Atlas (TCGA) [3]. The 10-fold cross-validation results for the training and testing datasets in that study demonstrated the excellent performance of the back propagation (BP) and learning vector quantization models in terms of prediction accuracy, area under the curve (AUC) values, robustness, accuracy, and sensitivity. Their results inspired the models developed in the current study, which integrate gene expression profiling data and artificial intelligence algorithms in improved diagnostic tools for CRC. In another recent study, accuracy in predicting breast cancer recurrence was compared among conventional and recently developed data mining algorithms [4]. According to the comparison results, the decision tree C5.0 algorithm may be the best tool for predicting breast cancer recurrence, particularly 3-year recurrence, in patients who are in distant recurrence stage or nonrecurrence stage. In the dataset, the best predictors of breast cancer recurrence were lymph node (LN) involvement, human epidermal growth factor-receptor-2 (HER2) value, tumor size, and tumor margin (free versus closed). In another recent study, a naïve Bayesian classifier (NBC) model were used to predict breast cancer recurrence within 5 years after breast cancer surgery. The prediction performance of the proposed NBC model was comparable to that of previous models that have used support vector machine (SVM) (AUC = 0.85) or ANN (AUC = 0.85) [5]. The nomogram-based approach is attractive because it does not require computation or calculation for prediction of breast cancer recurrence.

Although many forecasting models for predicting outcomes after breast cancer surgery have been proposed in recent years, models for predicting recurrence within 10 years after breast cancer surgery have had major shortcomings: (1) recently proposed forecasting models have lower prediction accuracy compared to conventional models [6,7], (2) proposed forecasting models require use of health insurance claims data, which may be unavailable for real-time use in clinical settings [8,9], and (3) predictions of postoperative recurrence after breast surgery do not consider demographic characteristics, clinical characteristics, quality of care and preoperative health-related quality of life [10,11]. Successful applications of statistical data mining and machine learning methods have been demonstrated in the medical field [7,8,9,10,11]. Clinical and genetic information can be used to improve precision in estimating prognosis and to obtain a comprehensive overview of a disease. Given the rapid accumulation of real-world data, the development of machine learning technologies provides the capability to generate risk stratification models that can efficiently consider numerous predictors.

Few studies of recurrence after breast cancer surgery have used longitudinal data for more than ten years. Moreover, no studies have considered group differences in factors other than outcome, such as demographic characteristics and clinical characteristics. Additionally, no studies have discussed machine learning algorithms for predicting recurrence within 10 years after breast cancer surgery. Health researchers can use the predictive simulation results obtained in this study not only to develop and improve healthcare policies, but also to improve healthcare decision making. The aim of this study was to compare the five forecasting models in terms of accuracy in predicting and identifying significant predictors of recurrence within 10 years after breast cancer surgery.

## 2. Materials and Methods

### 2.1. Study Design and Patients

**Figure 1:**
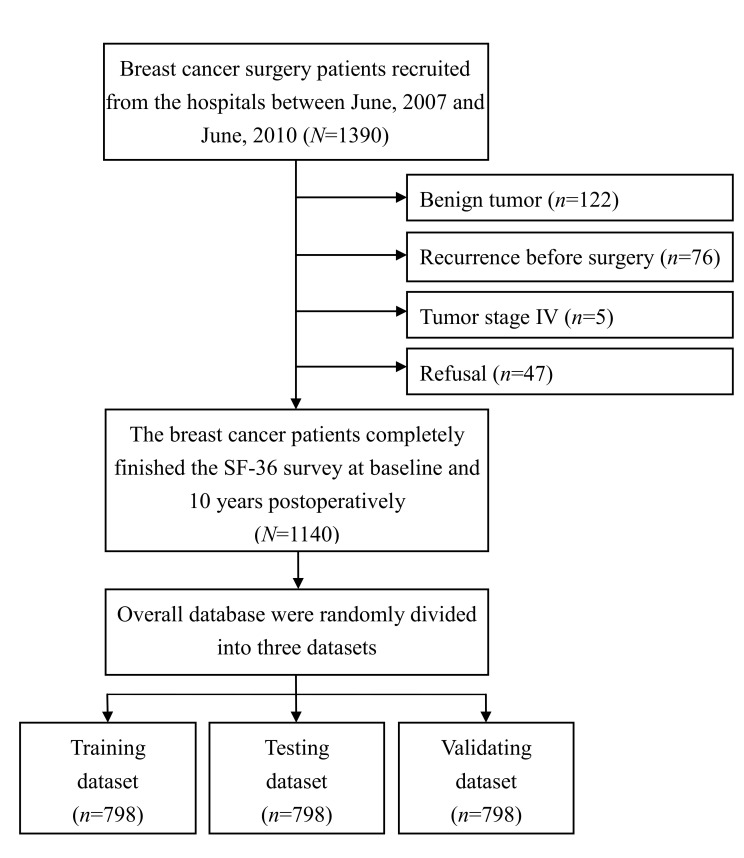
Flowchart of the study procedure.

The researchers in this prospective cohort study used structured questionnaires to interview the participants. This study recruited patients who had a primary diagnostic code for breast cancer (ICD-9-CM 174–174.9) and a history of breast cancer surgery performed at one of three medical centers in southern Taiwan between June 2007 and June 2010. Additional inclusion criteria were (1) history of no more than one surgical procedure for breast cancer; (2) history of breast conservation surgery (BCS), modified reconstructive mastectomy (MRM), or mastectomy with reconstruction; (3) clear consciousness and fluency in Chinese or Taiwanese; and (4) willingness to participate in interviews. The exclusion criteria were (1) benign tumor; (2) tumor stage IV; (3) cognitive impairment; and (4) refusal to participate. Of the patients who met the criteria for inclusion in the study, 1140 patients completed the written consent form and the SF-36 survey at baseline and at 10 years postoperatively. The average duration of follow up after breast cancer surgery was 10.8 years (Figure 1). The study protocol was approved by the institutional review board at Kaohsiung Medical University Hospital (KMUH-IRB-960186, date of approval: 20 June 2007), and written informed consent was obtained from each participant.

### 2.2. Statements of the Forecasting Models

#### 2.2.1. Artificial Neural Networks (ANN) Model

An ANN is a data processing algorithm in which the computations simulate a biological neural network [12]. An ANN model has three layers: an input layer, a hidden layer, and an output layer. Links connect nodes in different layers. Nodes in the input layer represent predictors, and nodes in the output layer represent outcomes. A common application of neural networks is the multilayer backpropagation learning algorithm, which models nonlinear systems. The present study used a multi-layer perceptron neural network (MLPNN). Despite the increased complexity of interpreting neural network outputs compared to outputs of other statistical models, the ANN model has been widely used in various medical fields [13,14].

#### 2.2.2. K-Nearest Neighbor (KNN) Model

The KNN algorithm classifies variables according to the closest training data in the feature space [15]. The KNN model uses instance-based learning method, which is among the simplest data mining algorithms, for majority voting on outcomes of points that are k-nearest to the new sample.

#### 2.2.3. Support Vector Machine (SVM) Model

The SVM is a supervised algorithm that divides the feature space into hyperplanes according to the target classes [16]. The SVM performs classification by maximizing the margin of the hyperplane that intercepts classes. This algorithm enhances classification accuracy by plotting a multidimensional hyperplane that divides classes and increases the margins between classes. The SVM also uses kernel functions to discriminate between nonlinearly separable classes.

#### 2.2.4. Naïve Bayesian Classifier (NBC) Model

An NBC assumes that the presence of a particular feature in a class is unrelated to the presence of any other feature [17]. Each feature is an independent and equal contributor to the outcome. The Bayes Theorem finds the probability of an event occurring given the probability of another event that has already occurred. An NBC model can be used for efficiently developing classification tools in various health domains and for transforming complex clinical problems into clear and precise predictive models.

#### 2.2.5. Cox Proportional-Hazards Regression (COX) Model

The COX proportional-hazards model, which is essentially a regression model, is widely used by medical researchers for statistical comparisons of variables as predictors of disease recurrence [18]. This semi parametric regression model can accommodate both discrete and continuous measures of event times.

### 2.3. Potential Predictors

A researcher collected the following data from medical records: demographic characteristics (age, education, current residence with other family members, marital status, body mass index, Charlson comorbidity index, tumor size, tumor stage, smoking, drinking, and breast cancer history), clinical characteristics (surgery type, American Society of Anesthesiologists score, chemotherapy, radiotherapy, hormonal therapy, surgeon volume, and hospital volume), quality of care within 10 years (readmission in 30 days, recurrence, and survival), and preoperative health-related quality of life (preoperative SF-36 Physical Component Summary (PCS) score and Mental Component Summary (MCS) score). The surgeons and hospitals included in the database were sorted by total numbers of surgical procedures, and each procedure was assigned a unique identification code. In accordance with outcome-volume studies performed earlier by the authors, surgeon who had performed ≤8, 9–16 and ≥17 cases/year were classified as low-, medium-, and high-volume surgeons, respectively, and hospitals that had performed ≤19, 20–29, and ≥30 cases/year were classified as low-, medium-, and high-volume hospitals, respectively [19,20]. The Chinese version of the SF-36 used in this study has been validated and widely used in both clinical practice and research [21]. To compare overall physical and mental functioning between the study population and the general Taiwan population, SF-36 PCS and MCS scores were calculated by norm-based scoring methods. As described in a previous study, the SF-36 PCS scores and MCS scores were converted to obtain means of 50 and standard deviations of 10 (compared to the “nationwide” normal group) [22].

### 2.4. Statistical Analysis

The unit of analysis in this study was the individual patient who had completed breast cancer surgery. Statistical analysis was performed in the following four steps. In the first step, which was performed before statistical analysis, the cases in the overall database were randomly divided into three datasets: a training dataset of 798 cases for model development, a testing dataset of 171 cases for internal validation, and a validating dataset of 171 cases for external validation. The independent variables fitted to the forecasting models were the significant predictors, and the dependent variable was the recurrence within 10 years after breast cancer surgery. After model training, model outputs were collected for each testing dataset. In the second step of statistical analysis, univariate Cox regression analyses were performed to identify significant predictors (*p* < 0.05). In comparisons of patient characteristics between the training dataset and the testing dataset, the statistical significance of continuous variables was tested by one-way analysis of variance, and the statistical significance of categorical variables was tested by Fisher exact analysis (*p* < 0.05). In the third step of the statistical analysis, 1000 pairs of forecasting models with 95% confidence intervals (95% CI) were compared in terms of accuracy in predicting recurrence within 10 years in breast cancer patients after surgery. The statistical significance of differences in performance indices between the two models was calculated by Chi-square test since this nonparametric test does not require a normal distribution of either the data or the variances [23]. Indices used for performance comparisons included sensitivity, specificity, positive and negative predictive value (PPV and NPV), accuracy, and area under the receiver operating characteristics (AUROC) curve. In the fourth and final step of statistical analysis, global sensitivity analysis was performed to assess the importance of variables in the forecasting model, to assess the relative significance of the predictors in the forecasting model, and to rank the importance of the predictors. The global sensitivity of the input variables against the output variable was expressed as the ratio of the network error (sum of squared residuals). Variables with a sensitivity ratio (VSR) of 1 or lower were assumed to diminish performance and were removed.

All statistical analyses were performed using the STATISTICA 13.0 software package (StatSoft, Inc., Tulsa, OK, USA). All statistical tests were two-sided; a *p* value less than 0.05 was considered statistically significant.

## 3. Results

### 3.1. Study Characteristics

Table 1 shows that the patients with breast cancer after surgery had a mean age of 52.30 years (standard deviation, SD 10.98 years) and that the largest proportion (40%) of patients was in tumor stage II. During the study period, 225 (19.7%) patients had recurrence within 10 years after breast cancer surgery. In the univariate Cox regression analysis, demographic characteristics, clinical characteristics, quality of care within 10 years, and preoperative health-related quality of life were significantly associated with recurrence within 10 years after breast cancer surgery (Table 2) (*p* < 0.05). Therefore, these predictors were included in the forecasting models.

### 3.2. Comparison of Forecasting Models

**Figure 2:**
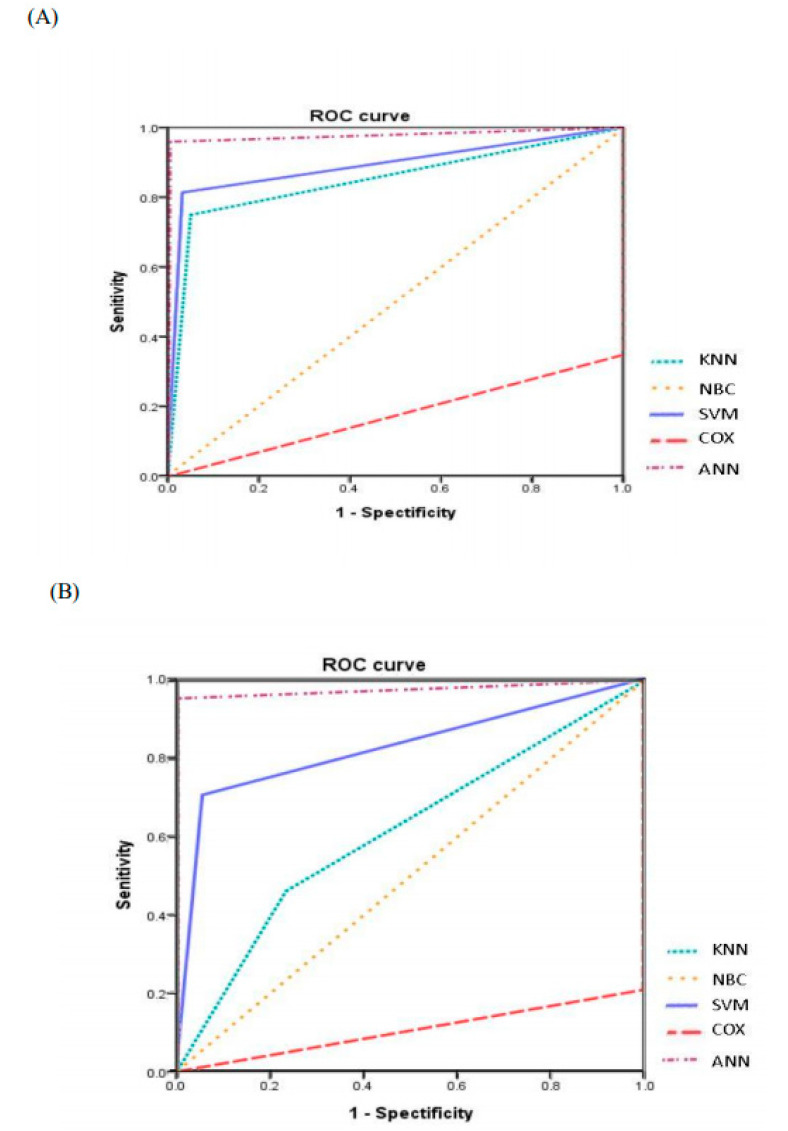
Receiver operating characteristics (ROC) curve for machine learning models in predicting recurrence within 10 years after breast cancer surgery in (A) training dataset and (B) testing dataset. (**A**) The comparison of the ROC curve between the forecasting models (artificial neural network (ANN), K nearest neighbor (KNN), support vector machine (SVM), naive Bayes classifier (NBC), and Cox regression (COX)) models to predict recurrence within 10 years after breast cancer surgery in the training dataset (*n* = 798). (**B**) The comparison of the ROC curve between the forecasting models to predict recurrence within 10 years after breast cancer surgery in the testing dataset (*n* = 171).

The training dataset and testing dataset did not significantly differ in patient characteristics, including recurrence within 10 years after breast cancer surgery (Table 3); therefore, samples were compared between the training dataset and testing dataset to increase the reliability of the validation results. In the current study, ANN model with 24 neurons in the input layer, 4 neurons in the hidden layer, and 1 neuron in the output layer. It showed that the ANN model performed significantly better in terms of sensitivity, specificity, PPV, NPV, accuracy, and AUROC values compared to the other forecasting models, and all differences were statistically significant (*p* < 0.001) (Table 4 and Figure 2).

### 3.3. Significant Predictors in the ANN Model

Next, the training dataset was used to calculate VSRs for the ANN model. The data in Table 5 indicate that, for predicting recurrence within 10 years after breast cancer surgery, global sensitivity analysis obtained the highest VSR (14.56; 95% CI 12.33–16.79) for surgeon volume, followed by hospital volume (VSR = 14.23; 95% CI 11.34–17.12) and tumor stage (VSR = 11.09; 95% CI 7.98–14.21). All VSR values for the ANN model exceeded 1, which indicated that network performance improved when all variables were considered.

### 3.4. Sensitivity Analysis

To verify the predictive accuracy of the models, this study also collected 171 additional datasets. Table 6 compares the performance indices values obtained by the ANN, KNN, SVM, NBC, and COX models for external validation. Again, the ANN model consistently obtained significantly better performance indices for predicting recurrence within 10 years after breast cancer surgery compared to the other forecasting models (*p* < 0.001).

## 4. Discussion

To the best of our knowledge, this study is the first to use forecasting models to analyze recurrence within 10 years after breast cancer surgery. Accuracy in predicting recurrence within 10 years in breast cancer patients after surgery was compared among the five forecasting models. When all models were constructed using a given set of clinical inputs, the ANN model was clearly superior to other forecasting models. Furthermore, unlike previous works in which the analyses were performed using a dataset for a single medical center, our study used prospective and longitudinal data from multiple medical centers, which provides a more accurate depiction of current treatment for breast cancer patients after surgery [7,8,9]. Additionally, in contrast with previous series studies that have used data for a single institution, this study used registry data to obtain a more accurate depiction of breast cancer surgery treatment in large populations. Using registry data also minimizes referral bias or bias caused by the practices of a single physician or a single institution [24].

Several strengths of this analysis should be noted. To our knowledge, this investigation is the first to compare machine learning algorithms, including regression-based method, to predict recurrence within 10 years after breast cancer surgery in a large general population. Unlike previously developed machine learning-based prognostic tools in oncology, the forecasting models in the study were trained on data for all patients treated at oncology or hematology/oncology clinics regardless of history of cancer-directed therapy. Furthermore, compared with machine learning algorithms previously applied in oncology, the forecasting models in this study included more numerous predictors, all of which are typically available in structured formats in real-time medical recorder databases. Thus, these forecasting models are more efficient than previously trained machine learning algorithms in the general oncology setting. The 10-year follow-up period in this prospective cohort study was also longer than that in previous works. Finally, most of the patients that the model classified as high-risk patients would be deemed appropriate for discussion of end-of-life preferences in a clinical setting.

Recent works have repeatedly demonstrated the superior performance of the ANN model compared to other forecasting models [25,26,27]. The advantages offered by the unique characteristics of the ANN model have been confirmed by statistical analyses. For example, using an ANN model enables more appropriate and more accurate processing of inputs that are incomplete or inputs that introduce noise. Another advantage is that linear and non-linear ANN models with good potential for use in large-scale medical databases can be constructed using data that are highly correlated but not normally distributed. Prognosis prediction is only one of the many applications of ANN models in clinical research in the medical field. Furthermore, the comparisons of various forecasting models in this study suggest that, by expanding the number of potential predictors, the ANN model facilitates systematic analysis of various diseases and facilitates comparisons of the effectiveness of research methods. Additionally, the proposed model can be extended to outcome prediction for treatments other than breast cancer surgery.

The global sensitivity analysis of the weights of significant predictors of recurrence within 10 years in breast cancer patients after surgery in this study revealed that the best predictor was surgeon volume, followed by hospital volume. This finding is consistent with earlier reports that, compared to all other breast cancer treatment variables, surgeon volume and hospital volume are the best predictors of breast cancer surgery outcomes, including treatment costs, health-related quality of life, readmission, complications, and recurrence after surgery [28,29,30]. Compared to a low-volume surgeon, a high-volume surgeon accompanied with a well-trained medical team tends to perform better in terms of operating time, quality of surgical procedure, discharge planning, and medical outcomes, all of which can potentially reduce postoperative recurrence. Morche et al. performed a meta-analysis of thirty-two reviews reporting on fifteen surgical procedures to investigate whether surgeon volume is a prognostic predictor of quality of health care [28]. Their meta-analysis of data for 32 publications with 15 different cancer procedures revealed that, in addition to volume-outcome relationship, surgeon volume is a significant independent predictor of medical outcomes in the general population of cancer patients.

Shi et al. retrospectively analyzed 97,215 breast cancer surgeries to examine the longitudinal effect of both hospital volume and surgeon volume on medical resource utilization and medical outcomes after surgical resection of breast cancer [21]. The study concluded that surgeon volume and hospital volume are significant independent predictors of total direct medical costs and postoperative recurrence (*p* < 0.001). The likely explanation for this finding is that ‘practice makes perfect’ and high surgical volumes not only improve surgical skills, but also reduce postoperative recurrence. The importance of surgeon volume and hospital volume for predicting outcomes in patients after cancer surgery is now well recognized. For investigators, these assessments enable a more comprehensive depiction of the potential burden on the patient after the envisaged (palliative) treatment in terms of its effects on medical resource utilization and medical outcomes simultaneously. Thus, surgeon volume and hospital volume, in addition to clinical attributes, should be included as a standard risk factor or predictor in future randomized controlled trials. Using s for stratification would improve the quality of future trials by increasing the homogeneity of treatment groups and would aid understanding of their results.

In agreement with previous studies [30,31], the present study found that advanced breast cancer stage was significantly associated with recurrence within 10 years after breast cancer surgery. During the study period, 369 patients (32.4%) had a tumor stage I, 456 (40.0%) tumor stage II, and 315 (27.6%) tumor stage III. Early diagnosis of breast cancer disease and curative retreatment are likely to improve recurrence. After surgery, breast cancer patients are often burdened by multiple cancer-related comorbidities that increase their risk of poor postoperative outcomes, including complications, a long hospital stay, a short survival time, and high treatment costs. As reported by Wu et al., tumor stage is an important predictor of recurrence after cancer surgery [32]. Our global sensitivity analysis also indicated that recurrence within 10 years after breast cancer surgery tends to increase in patients with late-stage tumors, which is consistent with other works [30,31,32].

This prospective observational study of a cohort of breast cancer surgery patients in Taiwan analyzed data for patients treated at multiple healthcare institutions. The ANN model developed in this study improves accuracy in identifying factors significantly associated with recurrence within 10 years after breast cancer surgery. However, the proposed forecasting model has many other potential clinical applications. For example, healthcare institutions can improve care quality by using the methods developed in this study to evaluate the effectiveness of medical treatment. Since the proposed ANN model accurately predicts recurrence within 10 years after breast cancer surgery, healthcare administrators and medical professionals at other institutions can use the model to demonstrate the need for prompt and appropriate postsurgical treatment. Broader potential applications of the model in Taiwan include facilitating the formulation and promotion of healthcare policies and the development of decision-support systems, which would ultimately contribute to improved health in all cancer patients. However, further studies are needed to determine the true clinical relevance of the ANN model and to clarify whether the model has practical clinical applications in predicting prognosis and in optimizing medical management for breast cancer patients after surgery.

This study has several limitations inherent in any large database analysis. First, the validity of the comparisons in the study is limited by the exclusion of complications associated with recurrence after surgery. Second, the analysis was limited to recurrence over a 10-year period after surgery, which reduced the subset of breast cancer patients in which the ANN model is clinically applicable. Third, this study only compared individual ANN, KNN, SVM, NBC, and COX models. Future works may consider the use of an alternative study design that compares a balanced sample of surgeons or hospitals at the first level and then randomly selects breast cancer patients at the second level. Thus, the relative importance of patient and provider characteristics could be delineated in multilevel modeling. Another advantage is that interacting effects of patient and provider characteristics on breast cancer recurrence could be detected. Nevertheless, the results can still be considered valid given the robustness and statistical significance of the results.

## 5. Conclusions

This study had two key findings: (1) the comparison of goodness-of-fit results indicated that provider characteristics (e.g., volume of breast cancer patients per surgeon and per hospital) are essential considerations in the design of clinical decision support systems; and (2) the comparison of AUROC values indicated that the ANN model is superior to other prediction models. Researchers can use the predictors identified by the ANN model in this study to educate patients who are candidates for breast cancer surgery or who have already received breast cancer surgery in the expected course of recovery and expected health outcomes. Integration of the machine learning algorithms applied in this study in other clinical decision-making tools would provide additional data that can be used to improve accuracy in predicting recurrence. Such data could be vital for developing, promoting, and improving health policies for treating breast cancer patients after surgery. Additionally, future research could explore designs for two-level or multi-level models that provide contextual effects of surgeon volume and hospital volume on breast cancer recurrence.

## Figures and Tables

**Table 1:** Characteristics of patients (*N* = 1140).

Variables	*N* (%)	Mean ± SD
Demographic characteristics		
Age, years		52.30 ± 10.98
Education, years		10.20 ± 3.77
Current residence with family member(s)	1095 (96.1%)	
Married	1002 (87.9%)	
Body mass index, kg/m^2^		24.55 ± 4.71
Charlson Comorbidity Index, score		1.05 ± 1.38
Tumor size		2.42 ± 1.80
Tumor stage		
I	369 (32.4%)	
II	456 (40.0%)	
III	315 (27.6%)	
Smoker	57 (5%)	
Drinker	30 (2.6%)	
Breast cancer history	153 (13.4%)	
Clinical characteristics		
Surgery		
BCS	117 (10.3%)	
MRM	306 (26.8%)	
Mastectomy with reconstruction	717 (62.9%)	
ASA score		2.04 ± 0.39
Chemotherapy	816 (71.6%)	
Radiotherapy	663 (58.2%)	
Hormonal therapy	681 (59.7%)	
Surgeon volume		
Low (≤8 cases/ year)	376 (33%)	
Medium (9 ~ 16 cases/ year)	381 (33.4%)	
High (≥17 cases/ year)	383 (33.6%)	
Hospital volume		
Low (≤19 cases/ year)	374 (32.8%)	
Medium (20~29 cases/ year)	381 (33.4%)	
High (≥30 cases/ year)	385 (33.8%)	
Quality of care within 10 years		
Readmission in 30 days	285 (25%)	
Recurrence	225 (19.7%)	
Survival	840 (73.7%)	
Preoperative quality of life		
Preoperative SF36 PCS score		56.02 ± 7.44
Preoperative SF36 MCS score		41.12 ± 18.39

BCS: breast conserving surgery; MRM: modified radical mastectomy; ASA: American Society of Anesthesiologists; PCS: physical component summary; MCS: mental component summary.

**Table 2:** Univariate Cox regression analysis of recurrence within 10 years after breast cancer surgery (*N* = 1140).

Variables	HR (95% CI)	*p* Value
Demographic characteristics		
Age, years	0.97 (0.97–0.98)	<0.001
Education, years	0.88 (0.87–0.90)	<0.001
Current residence with family member(s) (no vs. yes)	0.25 (0.22–0.29)	<0.001
Marital status (unmarried vs. married)	0.47 (0.44–0.51)	<0.001
Body mass index, kg/m^2^	0.95 (0.94–0.95)	<0.001
Charlson Comorbidity Index, score	0.62 (0.57–0.68)	<0.001
Tumor size, cm	0.66 (0.62–0.70)	<0.001
Tumor stage		
I vs. 0	0.19 (0.15–0.26)	< 0.001
II vs. 0	0.19 (0.15–0.24)	<0.001
≥III vs. 0	0.47 (0.36–0.62)	<0.001
Smoker (no vs. yes)	0.46 (0.26–0.81)	0.007
Drinker (no vs. yes)	0.11 (0.34–0.37)	<0.001
Breast cancer history (no vs. yes)	0.34 (0.24–0.49)	<0.001
Clinical characteristics		
Surgery type		
BCS	0.31 (0.16–0.63)	<0.001
MRM	2.00 (1.47–2.73)	<0.001
Mastectomy with reconstruction	0.75 (0.56–1.00)	0.050
ASA score	0.50 (0.47–0.54)	<0.001
Chemotherapy (no vs. yes)	0.28 (0.24–0.33)	<0.001
Radiotherapy (no vs. yes)	0.28 (0.23–0.33)	<0.001
Hormonal therapy (no vs. yes)	0.24 (0.20–0.30)	<0.001
Surgeon volume (medium vs. low)	0.98 (0.98–0.99)	<0.001
Surgeon volume (high vs. low)	0.97 (0.97–0.98)	<0.001
Hospital volume (medium vs. low)	0.98 (0.98–0.99)	<0.001
Hospital volume (high vs. low)	0.97 (0.97–0.98)	<0.001
Quality of care within 10 years		
Readmission in 30 days (no vs. yes)	0.23 (0.17–0.31)	<0.001
Postoperative reconstruction (no vs. yes)	0.50 (0.35–0.72)	<0.001
Preoperative quality of life		
Preoperative SF36 PCS score	0.97 (0.96–0.97)	<0.001
Preoperative SF36 MCS score	0.98 (0.97–0.98)	<0.001

HR: hazards ratio; BCS: breast conserving surgery; MRM: modified radical mastectomy; ASA: American Society of Anesthesiologists; PCS: physical component summary; MCS: mental component summary.

**Table 3:** Comparison of patient characteristics between training dataset and testing dataset.

Variables	Training Dataset (*n* = 798)	Testing Dataset (*n* = 171)	*p* Value
Demographic characteristics			
Age, years	51.97 ± 11.32	53.22 ± 10.94	0.189
Education, years	10.24 ± 3.83	10.13 ± 3.69	0.722
Current residence with family member(s)	770 (96.5%)	163 (95.3%)	0.462
Married	706 (88.5%)	150 (87.6%)	0.742
Body mass index, kg/m^2^	24.66 ± 4.92	24.36 ± 4.67	0.462
Charlson Comorbidity Index, score	1.05 ± 1.38	1.11 ± 1.35	0.656
Tumor size, cm	2.40 ± 1.82	2.56 ± 1.79	0.312
Tumor stage			0.052
I	271 (33.9%)	44 (25.7%)	
II	314 (39.3%)	75 (43.9%)	
≥III	213 (26.8%)	52 (30.4%)	
Smoker	36 (4.5%)	13 (7.6%)	0.094
Drinker	22 (2.8%)	6 (3.5%)	0.593
Breast cancer history	99 (12.4%)	32 (18.7%)	0.060
Clinical characteristics			
Surgery type			0.572
BCS	75 (9.4%)	19 (11.1%)	
MRM	218 (27.3%)	46 (26.9%)	
Mastectomy with reconstruction	505 (63.3%)	106 (62.0%)	
ASA score	2.04 ± 0.40	2.06 ± 0.35	0.399
Chemotherapy	565 (70.8%)	129 (75.4%)	0.237
Radiotherapy	464 (58.1%)	96 (56.1%)	0.581
Hormonal therapy	480 (60.2%)	94 (55.0%)	0.186
Surgeon volume			
Low	263 (33.0%)	56 (32.8%)	0.897
Medium	267 (33.4%)	57 (33.3%)	
High	268 (33.6%)	58 (33.9%)	
Hospital volume			
Low	262 (32.8%)	57 (33.3%)	0.796
Medium	266 (33.3%)	57 (33.3%)	
High	270 (33.8%)	57 (33.4%)	
Quality of care within 10 years			
Readmission in 30 days	203 (25.4%)	47 (27.6%)	0.551
Recurrence	147 (18.4%)	43 (25.3%)	0.057
Postoperative reconstruction	92 (11.5%)	20 (11.8%)	0.911
Preoperative quality of life			
Preoperative SF36 PCS score	56.19 ± 7.42	55.18 ± 7.86	0.112
Preoperative SF36 MCS score	41.16 ± 18.17	40.03 ± 19.46	0.468

BCS: breast conserving surgery; MRM: modified radical mastectomy; ASA: American Society of Anesthesiologists; PCS: physical component summary; MCS: mental component summary.

**Table 4:** Performance indices for machine learning models in predicting recurrence within 10 years after breast cancer surgery (unit: %).

Models	Sensitivity	Specificity	PPV	NPV	Accuracy	AUROC
Training dataset (*n* = 798)						
ANN	95.89 (94.68–97.09)	99.54 (99.43–99.66)	97.90 (96.04–99.75)	99.08 (98.90–99.26)	98.87 (98.12–99.63)	97.62 (96.87–98.37)
KNN	75.00 (71.67–78.34)	94.97 (92.58–97.36)	78.94 (75.12–82.76)	93.78 (92.67–94.89)	90.95 (89.47–92.44)	85.00 (83.78–86.23)
SVM	81.29 (80.04–82.57)	96.61 (95.49–97.73)	85.80 (83.78–87.82)	95.35 (94.22–96.48)	95.53 (94.87–96.19)	88.90 (86.76–91.04)
NBC	100.00 (99.91–100.00)	0.00 (0.00–0.00)	79.88 (76.47–83.29)	0.00 (0.00–0.00)	79.88 (76.23–83.53)	50.00 (42.65–57.36)
COX	34.93 (24.91–44.99)	0.00 (0.00–0.00)	7.30 (4.37–10.24)	0.00 (0.00–0.00)	6.42 (5.78–7.06)	17.50 (12.14–22.86)
*p* value	<0.001	<0.001	<0.001	<0.001	<0.001	<0.001
Testing dataset (*n* = 171)						
ANN	95.35 (94.47–96.23)	100.00 (99.94–100.00)	100.00 (99.97–100.00)	98.45 (97.87–99.03)	98.82 (97.67–99.96)	99.81 (99.43–99.99)
KNN	46.15 (36.21–56.09)	76.66 (73.69–79.63)	46.15 (35.98–56.32)	76.67 (72.41–80.93)	67.44 (55.67–79.21)	61.40 (47.93–74.87)
SVM	70.37 (65.45–75.29)	94.35 (93.35–95.37)	75.50 (71.41–79.59)	93.13 (91.47–94.79)	89.79 (87.77–91.80)	82.40 (80.34–84.46)
NBC	100.00 (99.91–100.00)	0.00 (0.00–0.00)	19.01 (10.98–27.04)	0.00 (0.00–0.00)	19.01 (10.23–27.79)	50.00 (40.67–59.34)
COX	20.93 (8.79–33.07)	0.00 (0.00–0.00)	6.62 (3.78–9.46)	0.00 (0.00–0.00)	5.29 (3.02–7.47)	10.50 (4.71–16.29)
*p* value	<0.001	<0.001	<0.001	<0.001	<0.001	<0.001

ANN: artificial neural network; KNN: k-nearest neighbor; SVM: support vector machine; NBC: naïve Bayesian classifier; PPV: positive predictive value; NPV: negative predictive value; AUROC: area under the receiver operating characteristic curve.* 1000 pairs of forecasting models with bootstrapping methods were compared in terms of accuracy in predicting recurrence within 10 years after breast cancer surgery.

**Table 5:** Global sensitivity analysis of artificial neural network model in predicting recurrence within 10 years after breast cancer surgery (*N* = 798).

	Rank 1st	Rank 2nd	Rank 3rd
Variable	Surgeon volume	Hospital volume	Tumor stage
VSR (95% CI)	14.56 (12.33–16.79)	14.23 (11.34–17.12)	11.09 (7.98–14.21)

VSR: Variable sensitivity ratio; PCS: physical component summary; MCS: mental component summary; CI: confidence interval.

**Table 6:** Performance indices of forecasting models when using the validating dataset (*n* = 171) to predict recurrence within 10 years after breast cancer surgery (unit: %) *_._

Models	Sensitivity	Specificity	PPV	NPV	Accuracy	AUROC
ANN	88.90	95.52	84.21	96.97	94.12	97.62
	(86.69–91.11)	(94.87–96.17)	(82.67–85.75)	(95.57–98.38)	(93.01–95.23)	(96.83–98.41)
KNN	46.15	76.66	46.15	76.67	67.44	61.40
	(35.14–57.16)	(71.48–81.84)	(35.24–57.06)	(65.69–87.65)	(61.12–73.76)	(55.41–67.39)
SVM	70.37	94.35	75.50	93.13	89.79	82.40
	(65.67–75.07)	(93.24–95.46)	(70.49–80.51)	(92.01–94.25)	(88.12–91.46)	(81.12–83.68)
NBC	100.00	0.00	19.01	0.00	19.01	50.00
	(99.94–100.00)	(0.00–0.00)	(9.98–28.04)	(0.00–0.00)	(9.57–28.45)	(39.68–60.32)
COX	20.93	0.60	6.62	0.23	5.29	10.50
	(7.93–33.95)	(0.00–1.01)	(4.32–8.92)	(0.10–0.36)	(3.37–7.21)	(4.78–16.22)
*p* value	<0.001	<0.001	<0.001	<0.001	<0.001	<0.001

ANN: artificial neural network; KNN: k-nearest neighbor; SVM: support vector machine; NBC: naïve Bayesian classifier; PPV: positive predictive value; NPV: negative predictive value; AUROC: area under the receiver operating characteristic curve. * 1000 pairs of forecasting models with bootstrapping methods were compared in terms of accuracy in predicting recurrence within 10 years after breast cancer surgery.
